# Proteomic profiling of the plasma of Gambian children with cerebral malaria

**DOI:** 10.1186/s12936-018-2487-y

**Published:** 2018-09-24

**Authors:** Ehab M. Moussa, Honglei Huang, Marie L. Thézénas, Roman Fischer, Abhinay Ramaprasad, Fatou Sisay-Joof, Muminatou Jallow, Arnab Pain, Dominic Kwiatkowski, Benedikt M. Kessler, Climent Casals-Pascual

**Affiliations:** 1 Wellcome Trust Centre for Human Genetics, Oxford, UK; 2 King Abdulla University of Science and Technology, Thuwal, Saudi Arabia; 3 MRC Laboratories, Banjul, The Gambia; 4 Hospital Clinic i Provincial de Barcelona, CDB and ISGlobal, Barcelona, Spain

**Keywords:** *Plasmodium falciparum*, Cerebral malaria, Coagulation, Acute phase reaction, Proteasome, Biomarkers

## Abstract

**Background:**

Cerebral malaria (CM) is a severe neurological complication of *Plasmodium falciparum* infection. A number of pathological findings have been correlated with pediatric CM including sequestration, platelet accumulation, petechial haemorrhage and retinopathy. However, the molecular mechanisms leading to death in CM are not yet fully understood.

**Methods:**

A shotgun plasma proteomic study was conducted using samples form 52 Gambian children with CM admitted to hospital. Based on clinical outcome, children were assigned to two groups: reversible and fatal CM. Label-free liquid chromatography–tandem mass spectrometry was used to identify and compare plasma proteins that were differentially regulated in children who recovered from CM and those who died. Candidate biomarkers were validated using enzyme immunoassays.

**Results:**

The plasma proteomic signature of children with CM identified 266 proteins differentially regulated in children with fatal CM. Proteins from the coagulation cascade were consistently decreased in fatal CM, whereas the plasma proteomic signature associated with fatal CM underscored the importance of endothelial activation, tissue damage, inflammation, haemolysis and glucose metabolism. The concentration of circulating proteasomes or PSMB9 in plasma was not significantly different in fatal CM when compared with survivors. Plasma PSMB9 concentration was higher in patients who presented with seizures and was significantly correlated with the number of seizures observed in patients with CM during admission.

**Conclusions:**

The results indicate that increased tissue damage and hypercoagulability may play an important role in fatal CM. The diagnostic value of this molecular signature to identify children at high risk of dying to optimize patient referral practices should be validated prospectively.

**Electronic supplementary material:**

The online version of this article (10.1186/s12936-018-2487-y) contains supplementary material, which is available to authorized users.

## Background

Severe malaria (SM) is a major public health problem that accounts for more than 400,000 deaths each year, mainly in sub-Saharan Africa [1]. Cerebral malaria (CM) is a severe neurological complication of malaria infection that causes acute non-traumatic encephalopathy. Children with CM usually present with coma and 1–4 days history of fever and convulsions [2]. In African children, petechial brain haemorrhage and retinopathy were found to correlate with fatality in children with CM [3]. The majority of the children who are admitted at an early stage of the disease and treated appropriately recover consciousness in 2–3 days. However, nearly one-fifth of these children develop neurological sequelae [2, 4].

The pathophysiology of CM is poorly understood and several mechanisms of disease have been proposed [5]. Parasite sequestration and cytokine activation are the two leading hypotheses to explain CM pathogenesis [2, 6]. Sequestration of parasite-infected erythrocytes (PfIEs) obstructs brain microvasculature causing hypoxia to the surrounding brain parenchyma and subsequent brain injury. Up-regulation of cytokines, mainly TNF in Gambian and Ghanaian children was also correlated with CM [7–9]. TNF induces iNOS, an enzyme which produces nitric oxide, a free radical that modifies brain signaling and electrolyte physiology [10].

The function of the vascular endothelium is intrinsically linked to the coagulation cascade, and any biological or pathological change of the former will necessarily disrupt the function of the latter. Indeed, dysregulation of the coagulation cascade has been observed in CM [11]. Upregulation of endothelial and platelet-derived micro-particles with increased adhesiveness of endothelial cell-leukocyte-platelet interaction was shown in children with CM. In addition, platelet adhesion and aggregation are involved in cytoadhesion of PfIEs [12, 13] and in modulating sequestration [14]. Furthermore, the severity of CM in children was correlated with the inhibition of ADAMTS13 and thus with an increase of abnormal circulating ultra large von Willebrand factor (ULVWF) multimers that cross-link platelets, induce thrombocytopaenia [5] and enhance obstruction of the brain microvasculature [15, 16]. More recently, the role of the endothelial protein C receptor (EPCR) has been reported as a major biological player in *Plasmodium falciparum* CM [17].

Tissue damage is an inevitable consequence of any severe infection, particularly when oxygen supply to peripheral tissues is compromised by vascular obstruction. The role of muscle damage during the course of CM has been recently inferred from a strong signature of muscle proteins in plasma from CM patients [18]. However, it remains unclear if this signature derives from microvascular lesions in the muscle or from muscle tissue damage associated with the seizing activity frequently observed in patients with CM [19]. High levels of 20S proteasomes in human serum, conventionally called circulating proteasomes, are increased in patients with conditions such as sepsis and malignancies [20], usually associated with tissue damage and muscle wasting. This process is critical in severe infection and other hypercatabolic states that result in high protein turnover [21].

Discovery proteomics using tandem mass spectrometry is increasingly being used to investigate changes in the proteome of specific physiological compartments, which often capture disease-specific changes of clinical relevance [22]. To better understand the disease mechanism associated with fatal CM and with a view to identify prognostic markers, a shotgun proteomic analysis was conducted to compare the plasma proteomic signature of children with CM that survived and those that died.

## Methods

### Clinical study

EDTA-plasma samples were collected from children aged 4 months to 14 years admitted to the Royal Victoria Teaching Hospital (RVTH) in Banjul, the main health facility in The Gambia, from January 1997 to December 2009 [23]. All patients included in the study had a blood smear positive for asexual *P. falciparum* parasites and met one or more of the WHO criteria for severe malaria [19]. Samples were collected before patients were transfused. Non-survivor patients are those who died within 48 h of admission. CM was defined as a Blantyre coma score of 2 or less with any *P. falciparum* parasite density. The coma score was carried out at least 30 min after the last seizure and after correction of hypoglycaemia, and at least 6 h after treatment of seizures with anticonvulsants.

### Sample preparation

Samples were selected and separated by phenotype (CM dead–CM alive) and three pools of samples per group were generated prior to analysis. The 14 most abundant human plasma proteins were depleted using the Agilent Multiple Affinity Removal Spin Cartridge System (Agilent) according to the manufacturer’s instructions. Briefly, human plasma samples were centrifuged at 12,000*g* for 10 min to remove any precipitates and particles, then 10 µl of plasma were further diluted with 200 µl of buffer A and filtered through 0.22-µm spin filter. The spin cartridge was flushed with 2 ml of Buffer A (Agilent), next 200 µl of diluted plasma sample were loaded to the cartridge and spun at 100×*g* for 1.5 min and the flow-through containing remaining plasma proteins was then collected. The cartridge was washed with 400 µl of Buffer A and spun at 100×*g* for 2.5 min. The wash step was repeated twice. Finally, the bound top 14 proteins were eluted by adding 2 ml of buffer B (Agilent) followed by re-equilibration with 4 ml of Buffer A before the next run.

Top 14-depleted plasma samples were further concentrated and desalted by TCA/DOC precipitation. Sodium deoxycholate (final concentration: 125 µg/ml) was added to the samples, vortexed, and left for 15 min at RT. Trichloroacetic acid (final concentration: 6%) was then added and the samples were centrifuged for 10 min at 12,000*g*. The pellet was washed in 100% ice-cold acetone and centrifuged at 10,000*g* for 5 min at 4 °C. The supernatant was discarded and the dried pellet was resuspended in 50 µl of buffer containing 6 M Urea and 100 mM Tris. Protein concentration was quantified using BCA assay (Thermo).

### SDS-PAGE and protein digestion

For each sample, 200 μg of proteins were resuspended in 200 μl with 1X Laemmli buffer and 20 μl loaded onto a criterion XT Bis–Tris gel 4–12% using 1X XT MES running buffer (Bio-Rad). The gels were stained with Instant Blue (Expedeon Ltd, Harston, UK) for 10 min and transferred in distilled water for direct use. For each sample, 15 bands were excised, washed in 50% ethanol 5% acetic acid, and dehydrated in acetonitrile (ACN). Gel pieces were treated with 10 mM DTT and 50 mM IAA, dehydrated with ACN, dried completely, rehydrated with 100 mM NH_4_HCO_3_ then dehydrated again and dried. Dried gel pieces were then incubated overnight at 37 °C with 60 ng trypsin (Promega) in 50 mM NH_4_HCO_3_. Peptides were then extracted with buffer B (85% ACN, 5% formic acid (FA)), dried down completely and resuspended in buffer A (2% ACN, 0.1% FA). Digested peptides were purified using SEP-PACK C18 column (Waters) and eluted with 0.6 ml buffer B (65% HPLC grade ACN and 0.1% FA) twice. Purified peptides were then completely vacuum dried in speed-vac (Thermo) and resuspended in 100 µl of Buffer A (2% ACN, 0.1% FA).

### Mass spectrometry analysis

Nano-LC–MS/MS analysis was performed using 100 µm-inner diameter × 10 cm C18 column (Proxeon) on a 90 min gradient of 2–42% solvent B (solvent A: 99.9% H_2_O, 0.1% FA; solvent B: 99.9% ACN, 0.1% FA). Two technical replicates per sample were analysed in positive mode using 1 μg of peptides per sample per run. The nano-LC system (final rate 0.25 μl/min) was coupled to a LTQ-Orbitrap Velos (Thermo) as described previously [24]. MS scans were performed with a mass range of 300–1600 (m/z) and a mass resolution of 60,000, analysing the top 20 precursor ions using collision induced dissociation (CID) mode in the Iontrap at collision energy of 35 V and dynamic peak exclusion duration of 20 s.

### Database searching and label- free protein quantitation

MS/MS spectra were extracted from raw files using Proteome Wizard MSConvert (Thermo) using the 200 most intense peaks in each spectrum and converted into MGF-format peaklists. The peaklists were searched against the “in-house” customized database for human plasma (human and *P. falciparum* sequences, humanIPI3.75_falciparum3D7) using the central proteomics facilities pipeline CPFP that combines data from three search engines (Mascot, OMSSA and X!tandem k-score) [25, 26]. Searches were performed using the following parameters: digestion enzyme, trypsin; fixed modification, carbamidomethylation of cysteine; variable modifications, oxidation of methionine and lysine and deamidation of asparagine and glutamine; peptide tolerance, 20 ppm; fragment tolerance, 0.5 Da; missed cleavages. (1) Similar proteins were grouped and only non-conflicting features (peptide spectrum matches) were used for quantitation. The label-free analysis was carried out using the normalized spectral index SINQ [25].

### Immunoassays

Plasma levels of circulating proteasomes (20S) and PSMB9 were measured using a double antibody sandwich Enzyme-Linked Immunosorbent Assay (Antibodies-online Inc.). Assays were performed according to manufacturer’s instructions.

## Results

### Identification of proteomic markers associated with fatal outcome in cerebral malaria

In this study, 52 Gambian children with CM were included. Of these, 26 died and 26 survived. The only clinical feature that was significantly different between the survivors of CM and those with a fatal outcome was the presence of respiratory distress in those who died (*P *= 0.002). Parasite density and haemoglobin concentration were higher in patients who died, but these differences were of borderline statistical significance. The main clinical features of the two groups are described in Table 1.

**Table 1:** Properties of the study population as reported/measured in individual cases

	Survived (N = 26)	Died (N = 26)	*P* value
Sex (male/female)	14/15	15/9	0.33
Age (median, IQR) months	60 (35–95)	48 (24–83)	0.32
Hb (mean, SD) g/dl	7.27 (1.93)	8.03 (2.15)	0.07
Respiratory distress^a^ (%)	24.1	66.6	0.002
Convulsions on admission (%)	75	69.2	0.77
Deep coma (%)^a^	31	54	0.08
Transfused (%)	37.9	45.8	0.56
*P. falciparum* (geo mean, 95% CI) parasites/µl	35,341 (18,759–66,581)	55,792 (28,797–108,095)	0.051
Protein biomarkers			
Circulating 20S proteasomes (ng/ml)	39.4 (19.6–49.4)	22.7 (16–34.5)	0.20
PSMB9 (ng/ml)	177 (129–267)	209 (161–271)	0.49

^a^Deep coma was defined as a Blantyre Coma score of 0 or 1. Respiratory distress was defined as the presence of deep breathing, irregular breathing, or chest in-drawing

**Fig. 1:**
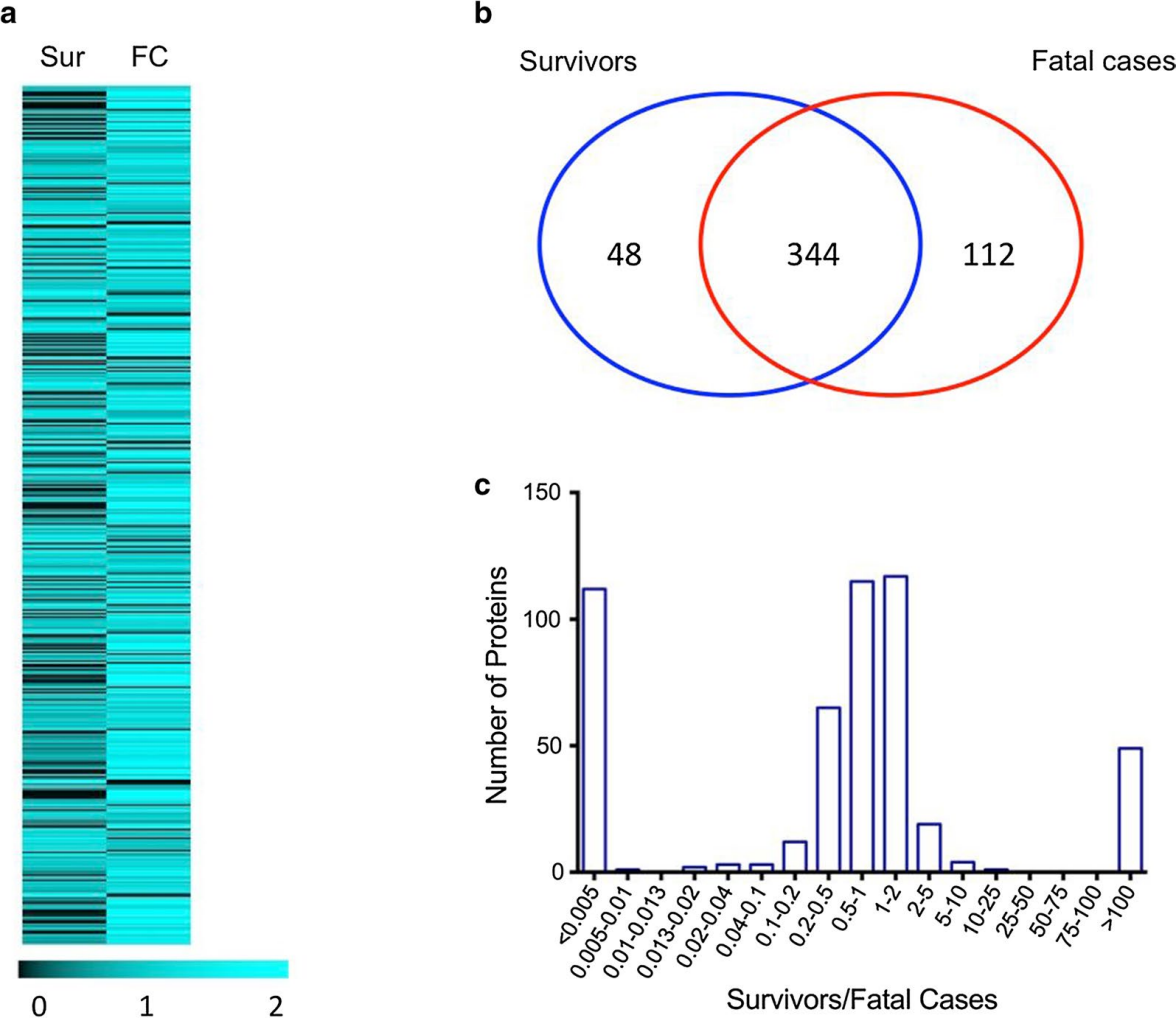
Plasma proteome profile of Gambian children with CM. **a** Heat map of the normalized protein quantitation value of detected plasma proteins according to the color scale at the bottom. Each row represents a protein. **b** Venn diagram indicates the total number of proteins identified and shows the unique proteins identified in pooled plasma samples of survivors and fatal cases and proteins identified in both groups. **c** Frequency distribution of the relative quantitation values of proteins in the plasma of survivors compared to fatal cases. *Sur* survivors, *FC* fatal cases

The plasma proteome of 52 Gambian children with CM was characterized using a shotgun proteomic approach. A total of 6296 peptides corresponding to 504 proteins were identified, 392 in children who survived and 456 in children who died (Fig. 1a, b). Accordingly, 266 proteins were identified as differentially expressed. Of these, 196 were up-regulated in children who died compared to 70 that were up-regulated in survivors (Fig. 1c).

### Host and parasite pathological modules associated with fatal outcome in cerebral malaria

**Fig. 2:**
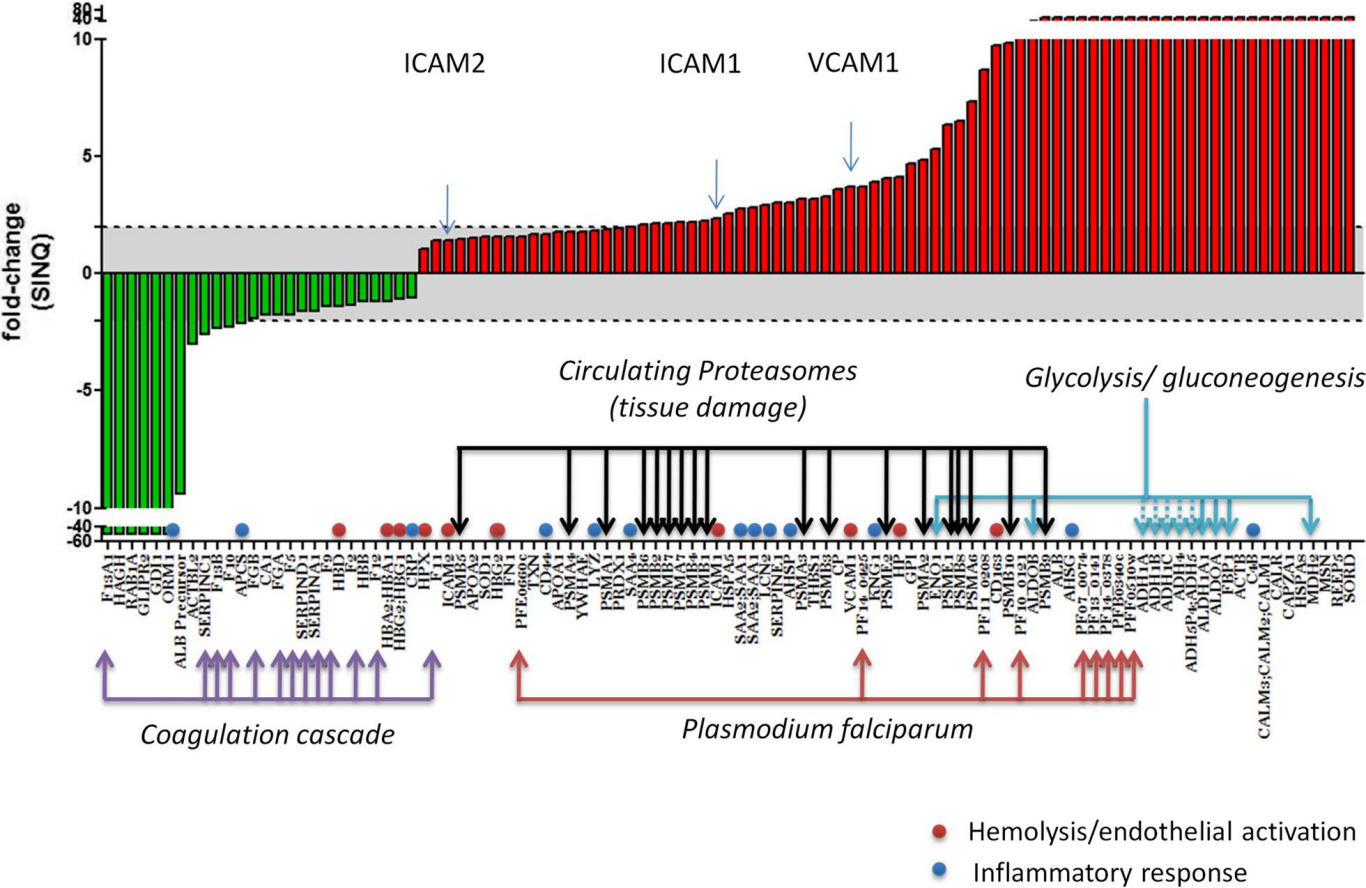
Proteomic signatures associated with pediatric fatal CM. Data show fold change in protein abundance as the ratio of proteomic quantification using SINQ values [25] in the fatal CM plasma pool to that in the reversible CM pool. Red indicates proteins increased in fatal CM cases and green indicates proteins increased in CM survivors. Dashed line indicates 1.5-fold-change in protein abundance

A complex proteomic signature was found to be associated with CM. A number of host and parasitic protein signatures were associated with a fatal outcome including endothelial activation, depletion of coagulation factors, activation of acute-phase reaction, glucose metabolism and tissue damage (represented by high levels of proteasome subunits) (Fig. 2 and Additional file 1). More specifically, these data confirm the role of three cell adhesion molecules (CAMs), VCAM-1, ICAM-1 and ICAM-2 in the pathophysiology of CM infection, all of them up-regulated in fatal cases. In addition, immunoglobulin J-chain and profilin-1, two of the ten most abundant microparticle (MP) proteins, were found to be decreased in fatal cases.

*Plasmodium falciparum* parasite proteins were also detected and all were consistently higher in fatal cases, probably indicating an association of poor clinical outcome with higher parasite densities and increased sequestration in brain microvasculature. Of these proteins, four enzymes are included in the glycolytic pathway, two enzymes in the purine salvage pathway, and one protein derives from the parasite’s cell membrane.

### High levels of 20S proteasome subunits and PSMB9 in plasma are associated with seizures in CM in children but not with death

A number of proteasome subunits were identified in patients with CM with a fatal outcome. It was hypothesized that this was consistent with an increase in circulating 20S proteasome subunits in fatal cases, probably resulting from ongoing cytolysis and tissue damage of undetermined origin (Fig. 2 and Additional file 1). However, the concentration of circulating proteasomes measured in individual samples was not significantly different between children with CM who survived and those who died (Additional file 2).

A similar analysis was then conducted measuring PSMB9 in individual patients because this marker showed the highest and most significant difference across groups. The concentration of PSMB9 was significantly different in patients with CM who died and those who survived. However, both circulating 20S proteasomes and PSMB9 were higher in those patients with CM who presented with convulsions during admission. Interestingly, PSMB9 was positively correlated with the number of witnessed seizure in patients with CM (r = 0.45, P = 0.01) (Additional file 2).

### Depletion of coagulation factors is associated with death

Of thirteen proteins involved in the coagulation cascade, eleven were down-regulated in fatal cases. Factors V and X, the main factors mediating prothrombin degradation on the activated platelet surface, were down-regulated in fatal cases. Of the intrinsic pathway proteins, factors IX and XII were down-regulated in fatal cases, whereas factor XI and kininogen-1 were up-regulated. Prothrombin (factor II) and fibrinogen were down regulated in fatal cases. Antithrombin III (Serpin C1) and heparin cofactor II (Serpin D1), both major thrombin inhibitors, were also down-regulated. Alpha-1 antitrypsin (Serpin A1: known to down-regulate factor XI) and plasminogen activator inhibitor-1 (PAI-1 or Serpin E1: inhibitor of tissue plasminogen activator and urokinase), were also decreased in fatal cases.

## Discussion

In this study, a comprehensive proteome characterization of plasma samples obtained from children with CM that survived is reported, and is compared with the signature of those who died. Here protein changes in three major pathobiological modules activated in fatal cases of CM is reported, namely (1) endothelial activation and inflammation, (2) circulating proteasomes possibly reflecting tissue damage and (3) depletion of proteins of the coagulation cascade. These biological signatures support current understanding of the pathogenesis of CM and show a novel association of the PSMB9 proteasome protein with the number of seizures witnessed in patients admitted to hospital with CM.

The findings in this study support the critical role of the vascular system in the pathogenesis of CM. During *P. falciparum* malaria infection, coagulation is activated as a result of cytokine-mediated endothelial activation, increased circulating endothelial and platelet-derived microparticles, and/or platelet adhesion to endothelium [11]. TNF-mediated upregulation of endothelial ICAM-1 enhances platelet adhesion to brain endothelial cells. Experimental models showing that ICAM-1 knockout mice are resistant to CM also support this finding [27]. In this study, ICAM-1, ICAM-2 and VCAM-1 were up-regulated in fatal cases, indicating that endothelial activation is associated with poor clinical outcome.

Vascular activation and inflammation are biologically linked and inflammatory cytokines are a key player. Indeed, CM has been associated with high concentrations of IL-6, IL-10, and TNF in plasma [2]. However, these cytokines circulate at very low concentrations (pg/ml to low ng/ml) in plasma. Despite extensive depletion of highly abundant proteins in combination with protein gel fractionation, our study failed to identify and quantify low abundant cytokines. Based on previous MS/MS quantification studies of *P. falciparum* proteins in these plasma samples [28], it is estimated that the LC–MS/MS workflow used here can identify and quantify proteins that circulate at concentrations of 100 ng/ml or higher. Therefore, low abundance cytokines like TNF, IL-10 or gamma-interferon could not be identified. Similarly, other important molecules like angiopoietins could not be identified in this study. The role of angiopoietins in the pathogenesis of CM has been reported both in children and adults with CM. These proteins capture disruptive changes of the vascular homeostasis which possibly result from *P. falciparum*-induced changes in endothelial cells. Indeed, the ratio of angiopoietin-1 to angiopoietin-2 has been proposed as a potential marker of severe malaria with prognostic value [29, 30]. The concentration of angiopoietins in plasma of CM patients is typically lower than 100 ng/ml, below the concentration range of proteins susceptible to be identified by our shotgun proteomic approach. Although the study was limited in the proteomic dynamic range explored using LC–MS/MS, other inflammatory mediators such as acute-phase proteins and complement were prominent in the signature of children with CM who died.

**Fig. 3:**
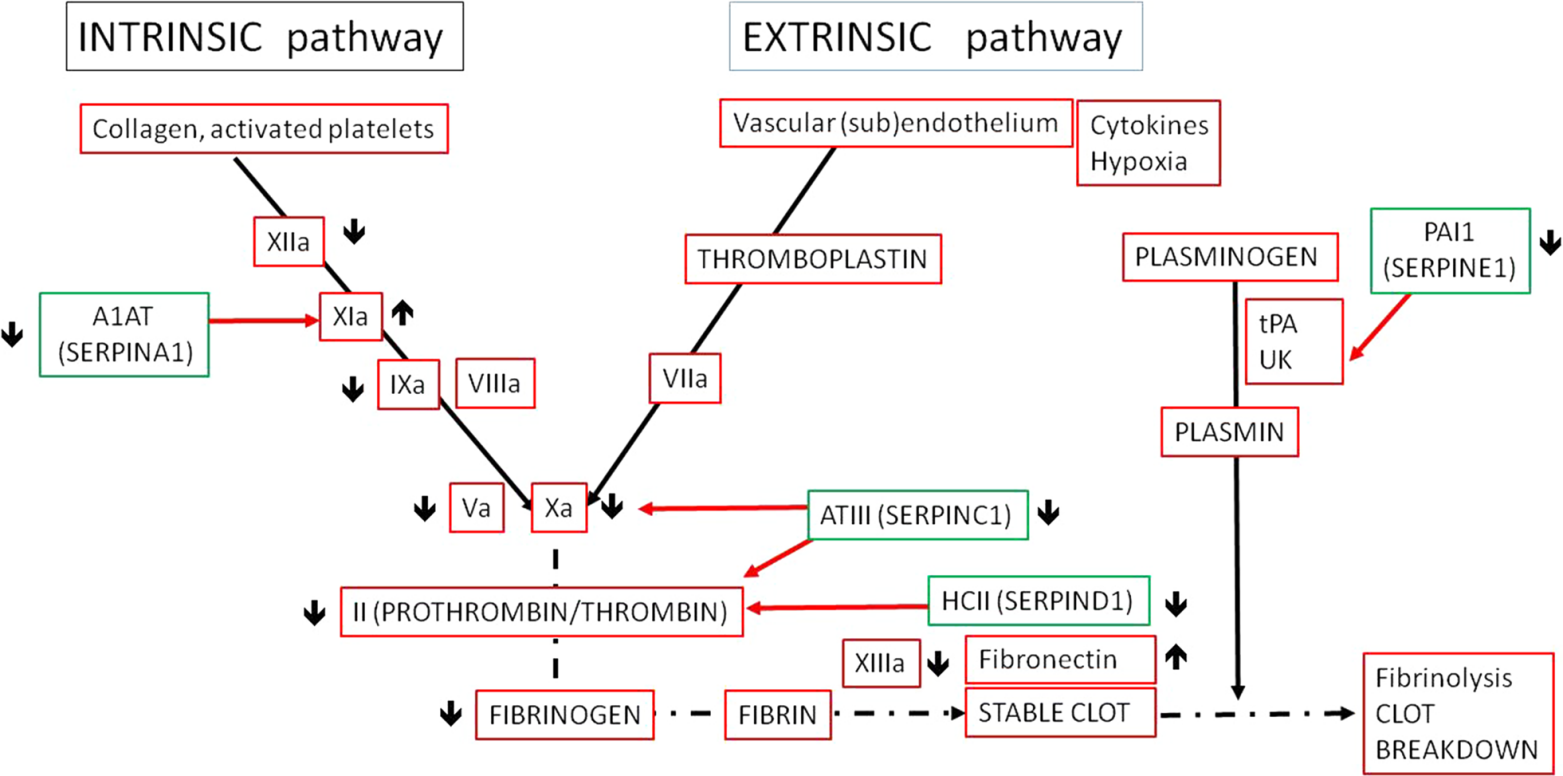
Coagulation cascade in CM. Data show differential regulation of proteins involved in the coagulation cascade (intrinsic and extrinsic pathways) in plasma from patients with fatal CM

The role of the coagulation cascade in the context of endothelial activation has acquired increasing importance in the pathogenesis of CM in recent years. Increased circulatory levels of the glycoprotein von Willebrand factor (vWF) has been associated with fatal CM, usually accompanied by a significant reduction of the plasma ADAMTS13 enzyme [11, 16]. *Plasmodium falciparum* parasites causing severe malaria have a stronger affinity for endothelial protein C receptor (EPCR) than parasites from children with uncomplicated malaria. Consequently, malaria-induced decrease of EPCR impairs the protein C system causing a sustained pro-coagulant state in the brain microvasculature [17, 31]. The data support a non-specific consumption and consequent depletion of coagulation proteins in children with CM who died compared with those that survived (outlined in Fig. 3). The majority of depleted coagulation factors that were identified belonged to the intrinsic pathway, which is triggered by endothelial activation. Similarly, downstream coagulation factors leading to clot formation (prothrombin and fibrinogen) and thrombin inhibitors like antithrombin-III (AT-III, SerpinC1) and heparin cofactor II (Serpin D1) were also decreased in fatal cases. Although these findings are compatible with a systemic activation of the coagulation system and possibly with disseminated intravascular coagulation, this diagnosis could not be formally ascertained in these patients.

Tissue damage is a prominent feature of CM and any other infection severe enough to compromise vascular flow and consequently oxygen delivery to cells. The role of muscle damage has been recently highlighted in a prospective study of CM in Nigerian children [18]. Therefore, it was hypothesized that the detection of increased levels of proteasomal proteins in plasma was possibly attributable to cell lysis in response to tissue hypoxia. Notably, all 20S proteasome subunits were consistently elevated in the plasma of fatal cases when compared with survivors. Circulating 20S proteasomes levels have been shown to increase in the sera of septic patients and in a number of haematopoietic and solid tumours indicating tissue damage [20, 32]. Erythrocytes are another potential source of human 20S proteasomes [33]. Consequently, ongoing haemolysis induced by red cell lyses of parasitized cells could account for this observation. However, haemolysis induces a major depletion of haptoglobin from plasma, which was not observed in fatal cases. It was, therefore, concluded that red cells were an unlikely source of proteasomes in these patients.

In contrast, total circulating proteasomes as measured by ELISA were higher in patients that survived. While these could be “transition states” towards forming the intact 20S, to the best of the authors knowledge it is not clear whether individual subunits may have distinct biological roles. Indeed, 20S proteasomes and PSMB9 were higher in those children with CM who presented with convulsions during admission to hospital. Interestingly, PSMB9 but not circulating 20S proteasomes were significantly correlated with the number of seizures witnessed during hospital admission. The biological significance of these finding remains unclear. The authors speculate that these proteasomes might originate from tissue damage that results from acute muscle contraction during seizing activity in children with CM. This hypothesis should be tested in prospective clinical studies for confirmation.

## Conclusion

An unbiased plasma proteomic study was conducted to identify proteins associated with a poor outcome in CM. This study not only supports the role of endothelial activation, tissue damage and the coagulation cascade in the pathogenesis of CM, but also highlights the prognostic importance of these pathobiological modules. In addition, these data show the association of higher levels of circulating proteasomes with seizures. The origin of these proteasomal proteins and their clinical significance are currently under study.

## Additional files

**Additional file 1.** List of differentially expressed proteins in fatal CM cases as measured by LC-MS/MS analysis.

**Additional file 2.** Circulating proteasomes and PSMB9 in patients with cerebral malaria. Dot plots show the concentration of total circulating 20S proteasomes (left panels) and PSMB9 (right panels) in children with CM based on outcome (top), presence of seizures during admission (middle) and the correlation of these biomarkers with the number of seizures witnessed during admission (bottom).

## References

[1] WHO. World Malaria Report 2016. Geneva: World Health organization; 2016.

[2] Idro R, Jenkins NE, Newton CR (2005). Pathogenesis, clinical features, and neurological outcome of cerebral malaria. Lancet Neurol..

[3] White VA, Lewallen S, Beare N, Kayira K, Carr RA, Taylor TE (2001). Correlation of retinal haemorrhages with brain haemorrhages in children dying of cerebral malaria in Malawi. Trans R Soc Trop Med Hyg.

[4] Newton CR, Hien TT, White N (2000). Cerebral malaria. J Neurol Neurosurg Psychiatry.

[5] van der Heyde HC, Nolan J, Combes V, Gramaglia I, Grau GE (2006). A unified hypothesis for the genesis of cerebral malaria: sequestration, inflammation and hemostasis leading to microcirculatory dysfunction. Trends Parasitol..

[6] Turner G (1997). Cerebral malaria. Brain Pathol.

[7] Akanmori BD, Kurtzhals JA, Goka BQ, Adabayeri V, Ofori MF, Nkrumah FK (2000). Distinct patterns of cytokine regulation in discrete clinical forms of *Plasmodium falciparum* malaria. Eur Cytokine Netw.

[8] Kwiatkowski D (1990). Tumour necrosis factor, fever and fatality in falciparum malaria. Immunol Lett.

[9] Kwiatkowski D, Hill AV, Sambou I, Twumasi P, Castracane J, Manogue KR (1990). TNF concentration in fatal cerebral, non-fatal cerebral, and uncomplicated *Plasmodium falciparum* malaria. Lancet.

[10] Clark IA, Cowden WB (2003). The pathophysiology of falciparum malaria. Pharmacol Ther.

[11] Moxon CA, Heyderman RS, Wassmer SC (2009). Dysregulation of coagulation in cerebral malaria. Mol Biochem Parasitol.

[12] Grau GE, Mackenzie CD, Carr RA, Redard M, Pizzolato G, Allasia C (2003). Platelet accumulation in brain microvessels in fatal pediatric cerebral malaria. J Infect Dis.

[13] Wassmer SC, Lepolard C, Traore B, Pouvelle B, Gysin J, Grau GE (2004). Platelets reorient *Plasmodium falciparum*-infected erythrocyte cytoadhesion to activated endothelial cells. J Infect Dis.

[14] Schofield L, Grau GE (2005). Immunological processes in malaria pathogenesis. Nat Rev Immunol.

[15] Larkin D, de Laat B, Jenkins PV, Bunn J, Craig AG, Terraube V (2009). Severe *Plasmodium falciparum* malaria is associated with circulating ultra-large von Willebrand multimers and ADAMTS13 inhibition. PLoS Pathog.

[16] Hollestelle MJ, Donkor C, Mantey EA, Chakravorty SJ, Craig A, Akoto AO (2006). von Willebrand factor propeptide in malaria: evidence of acute endothelial cell activation. Br J Haematol.

[17] Turner L, Lavstsen T, Berger SS, Wang CW, Petersen JE, Avril M (2013). Severe malaria is associated with parasite binding to endothelial protein C receptor. Nature.

[18] Bachmann J, Burte F, Pramana S, Conte I, Brown BJ, Orimadegun AE (2014). Affinity proteomics reveals elevated muscle proteins in plasma of children with cerebral malaria. PLoS Pathog.

[19] World Health Organization (2000). Communicable Diseases Cluster. Severe falciparum malaria. Trans R Soc Trop Med Hyg.

[20] Lavabre-Bertrand T, Henry L, Carillo S, Guiraud I, Ouali A, Dutaud D (2001). Plasma proteasome level is a potential marker in patients with solid tumors and hemopoietic malignancies. Cancer.

[21] Bhattacharyya S, Yu H, Mim C, Matouschek A (2014). Regulated protein turnover: snapshots of the proteasome in action. Nat Rev Mol Cell Biol.

[22] Anderson NL, Anderson NG (2002). The human plasma proteome: history, character, and diagnostic prospects. Mol Cell Proteomics.

[23] Jallow M, Casals-Pascual C, Ackerman H, Walther B, Walther M, Pinder M (2012). Clinical features of severe malaria associated with death: a 13-year observational study in the Gambia. PLoS One.

[24] Fischer R, Trudgian DC, Wright C, Thomas G, Bradbury LA, Brown MA (2012). Discovery of candidate serum proteomic and metabolomic biomarkers in ankylosing spondylitis. Mol Cell Proteomics.

[25] Trudgian DC, Ridlova G, Fischer R, Mackeen MM, Ternette N, Acuto O (2010). Comparative evaluation of label-free SINQ normalized spectral index quantitation in the central proteomics facilities pipeline. Proteomics.

[26] Trudgian DC, Thomas B, McGowan SJ, Kessler BM, Salek M, Acuto O (2010). CPFP: a central proteomics facilities pipeline. Bioinformatics.

[27] Favre N, Da Laperousaz C, Ryffel B, Weiss NA, Imhof BA, Rudin W, Lucas R, Piguet PF (1999). Role of ICAM-1 (CD54) in the development of murine cerebral malaria. Microbes Infect.

[28] Thezenas ML, Huang H, Njie M, Ramaprasad A, Nwakanma DC, Fischer R (2013). PfHPRT: a new biomarker candidate of acute *Plasmodium falciparum* infection. J Proteome Res.

[29] Conroy AL, Lafferty EI, Lovegrove FE, Krudsood S, Tangpukdee N, Liles WC (2009). Whole blood angiopoietin-1 and -2 levels discriminate cerebral and severe (non-cerebral) malaria from uncomplicated malaria. Malar J..

[30] Lovegrove FE, Tangpukdee N, Opoka RO, Lafferty EI, Rajwans N, Hawkes M (2009). Serum angiopoietin-1 and -2 levels discriminate cerebral malaria from uncomplicated malaria and predict clinical outcome in African children. PLoS One.

[31] Moxon CA, Wassmer SC, Milner DA, Chisala NV, Taylor TE, Seydel KB (2013). Loss of endothelial protein C receptors links coagulation and inflammation to parasite sequestration in cerebral malaria in African children. Blood.

[32] Roth GA, Moser B, Krenn C, Roth-Walter F, Hetz H, Richter S (2005). Heightened levels of circulating 20S proteasome in critically ill patients. Eur J Clin Invest.

[33] Claverol S, Burlet-Schiltz O, Girbal-Neuhauser E, Gairin JE, Monsarrat B (2002). Mapping and structural dissection of human 20 S proteasome using proteomic approaches. Mol Cell Proteomics.

