# Supplementary material for: Proteomic profiling of the plasma of Gambian children with cerebral malaria
Source: Malar J. 2018 Sep 24;17:337. doi: 10.1186/s12936-018-2487-y (PMC6154937; doi:10.1186/s12936-018-2487-y)
Supplement: Supplementary file 1 — Additional file 1. List of differentially expressed proteins in fatal CM cases as measured by LC-MS/MS analysis. [file 12936_2018_2487_MOESM1_ESM.docx]

**Additional File 1:** List of differentially expressed proteins in fatal CM cases as measured by LC-MS/MS analysis.

| Protein ID | Protein name | Sequence  coverage | | Unique  peptides | | Fold  Change § | Category |  |  |
| --- | --- | --- | --- | --- | --- | --- | --- | --- | --- |
| IPI00022429 | orosomucoid 1 | 15.9 | 3 | | -10 | | Acute-phase protein | |  |
| IPI00022391 | serum amyloid P component | 34.1 | 11 | | -2.13 | | Acute-phase protein | | |
| IPI00022389 | C-reactive protein | 36.2 | 12 | | -1.04 | | Acute-phase protein | | |
| IPI00022488 | hemopexin | 67.3 | 29 | | 1.05 | | Acute-phase protein | | |
| IPI00021854 | apolipoprotein A-II | 69 | 8 | | 1.50 | | Acute-phase protein | | |
| IPI00218733 | Soluble superoxide dismutase 1 | 67.5 | 6 | | 1.56 | | Acute-phase protein | | |
| IPI00845263 | fibronectin 1 | 52.3 | 89 | | 1.57 | | Acute-phase protein | | |
| IPI00021841 | apolipoprotein A-I | 61.8 | 18 | | 1.77 | | Acute-phase protein | | |
| IPI00019399 | serum amyloid A4 | 38.5 | 5 | | 2.00 | | Acute-phase protein | | |
| IPI00006146 | serum amyloid A2 | 58.2 | 7 | | 2.78 | | Acute-phase protein | | |
| IPI00552578 | serum amyloid A1 | 66.4 | 7 | | 2.79 | | Acute-phase protein | | |
| IPI00299547 | lipocalin 2 | 55.6 | 10 | | 2.91 | | Acute-phase protein | | |
| IPI00794184 | ceruplasmin | 73.4 | 51 | | 3.61 | | Acute-phase protein | | |
| IPI00745872 | albumin | 60.3 | 32 | | 40 | | Acute-phase protein | | |
| IPI00795830 | alpha-2-HS-glycoprotein | 55.6 | 10 | | 40 | | Acute-phase protein | | |
| IPI00297550 | coagulation factor XIII, A1 | 5.1 | 3 | | -20 | | Coagulation factor | | |
| IPI00032179 | serpin peptidase inhibitor, clade C | 68.1 | 37 | | -2.58 | | Coagulation factor | | |
| IPI00007240 | coagulation factor XIII, B | 38.7 | 19 | | -2.32 | | Coagulation factor | | |
| IPI00019576 | coagulation factor X | 32.6 | 14 | | -2.28 | | Coagulation factor | | |
| IPI00298497 | fibrinogen beta chain | 71.7 | 35 | | -1.93 | | Coagulation factor | | |
| IPI00021885 | fibrinogen alpha chain | 50.5 | 44 | | -1.77 | | Coagulation factor | | |
| IPI00022937 | coagulation factor V | 17.4 | 31 | | -1.74 | | Coagulation factor | | |
| IPI00292950 | heparin cofactor 2 | 57.5 | 24 | | -1.59 | | Coagulation factor | | |
| IPI00553177 | serpin peptidase inhibitor, clade A | 28 | 10 | | -1.58 | | Coagulation factor | | |
| IPI00296176 | coagulation factor IX | 41.6 | 14 | | -1.40 | | Coagulation factor | | |
| IPI00019568 | coagulation factor II (thrombin) | 58 | 33 | | -1.32 | | Coagulation factor | | |
| IPI00019581 | coagulation factor XII | 34 | 16 | | -1.16 | | Coagulation factor | | |
| IPI00008556 | coagulation factor XI | 11.7 | 6 | | 1.39 | | Coagulation factor | | |
| IPI00007118 | serpin peptidase inhibitor, clade E | 21.9 | 7 | | 3.00 | | Coagulation factor | | |
| IPI00032328 | kininogen 1 | 50.6 | 33 | | 3.92 | | Coagulation factor | | |
| IPI00009477 | intercellular adhesion molecule 2 | 12 | 3 | | 1.43 | | Endothelial activation | | |
| IPI00008494 | intercellular adhesion molecule 1 | 11.2 | 4 | | 2.35 | | Endothelial activation | | |
| IPI00018136 | vascular cell adhesion molecule 1 | 18 | 11 | | 3.69 | | Endothelial activation | | |
| IPI00027497 | glucose-6-phosphate isomerase | 20.7 | 10 | | 4.68 | | Glycolytic enzyme | | |
| IPI00465248 | enolase 1, (alpha) | 27.6 | 9 | | 5.33 | | Glycolytic enzyme | | |
| IPI00218407 | aldolase B, fructose-bisphosphate | 78 | 22 | | 33.89 | | Glycolytic enzyme | | |
| IPI00218896 | alcohol dehydrogenase 1A | 29.3 | 10 | | 40 | | Glycolytic enzyme | | |
| IPI00473031 | alcohol dehydrogenase 1B | 37.9 | 11 | | 40 | | Glycolytic enzyme | | |
| IPI00465343 | alcohol dehydrogenase 1C | 30.4 | 8 | | 40 | | Glycolytic enzyme | | |
| IPI00218899 | alcohol dehydrogenase 4 | 15 | 4 | | 40 | | Glycolytic enzyme | | |
| IPI00746777 | alcohol dehydrogenase 5 | 12.6 | 4 | | 40 | | Glycolytic enzyme | | |
| IPI00218914 | aldehyde dehydrogenase 1 | 24.8 | 8 | | 40 | | Glycolytic enzyme | | |
| IPI00796333 | aldolase A, fructose-bisphosphate | 51.7 | 21 | | 40 | | Glycolytic enzyme | | |
| IPI00073772 | fructose-1,6-bisphosphatase 1 | 42.9 | 11 | | 40 | | Glycolytic enzyme | | |
| IPI00473011 | hemoglobin, delta | 95.9 | 16 | | -1.37 | | Haemoglobin | | |
| IPI00654755 | hemoglobin, beta | 95.9 | 19 | | -1.20 | | Haemoglobin | | |
| IPI00410714 | hemoglobin, alpha 2 | 95.1 | 15 | | -1.16 | | Haemoglobin | | |
| IPI00220706 | hemoglobin, gamma G | 89.8 | 13 | | -1.07 | | Haemoglobin | | |
| IPI00554676 | hemoglobin subunit gamma-2 | 89.8 | 13 | | 1.57 | | Haemoglobin | | |
| IPI00010257 | alpha hemoglobin stabilizing protein | 45.1 | 5 | | 3.02 | | Haemoglobin | | |
| IPI00104074 | CD163 | 17.5 | 15 | | 9.71 | | Haemoglobin | | |
| PFE0660c | purine nucleotide phosphorylase | 18.8 | 4 | | 1.59 | | Parasite protein | | |
| PF14_0425 | fructose-bisphosphatealdolase | 42.8 | 13 | | 3.69 | | Parasite protein | | |
| PF11_0208 | phosphoglyceratemutase | 20.4 | 5 | | 8.71 | | Parasite protein | | |
| PF10_0121 | hypoxanthine phosphoribosyltransferase | 30.3 | 6 | | 26.62 | | Parasite protein | | |
| PF07_0074 | putative uncharacterized protein | 0.5 | 2 | | 40 | | Parasite protein | | |
| PF13_0141 | L-lactate dehydrogenase | 11.7 | 3 | | 40 | | Parasite protein | | |
| PF14_0378 | triosephosphateisomerase | 13.7 | 3 | | 40 | | Parasite protein | | |
| PFB0340c | serine-repeat antigen protein | 3.9 | 4 | | 40 | | Parasite protein | | |
| PFF0510w | histone H3 | 16.9 | 3 | | 40 | | Parasite protein | | |
| IPI00178926 | immunoglobulin J polypeptide | 15.7 | 3 | | -20 | | Microparticle protein | | |
| IPI00216691 | profilin 1 | 43.6 | 6 | | -1.68 | | Microparticle protein | | |
| IPI00479306 | proteasome subunit, beta type, 5 | 33.8 | 9 | | 1.47 | | Proteasome subunit | | |
| IPI00299155 | proteasome subunit, alpha type, 4 | 42.1 | 9 | | 1.78 | | Proteasome subunit | | |
| IPI00016832 | proteasome subunit, alpha type, 1 | 51.3 | 14 | | 1.88 | | Proteasome subunit | | |
| IPI00000811 | proteasome subunit, beta type, 6 | 37.7 | 7 | | 2.07 | | Proteasome subunit | | |
| IPI00028006 | proteasome subunit, beta type, 2 | 29.9 | 7 | | 2.13 | | Proteasome subunit | | |
| IPI00003217 | proteasome subunit, beta type, 7 | 16.6 | 4 | | 2.16 | | Proteasome subunit | | |
| IPI00024175 | proteasome subunit, alpha type, 7 | 45.6 | 10 | | 2.18 | | Proteasome subunit | | |
| IPI00555956 | proteasome subunit, beta type, 4 | 25 | 5 | | 2.19 | | Proteasome subunit | | |
| IPI00025019 | proteasome subunit, beta type, 1 | 38.6 | 8 | | 2.26 | | Proteasome subunit | | |
| IPI00171199 | proteasome subunit, alpha type, 3 | 35.9 | 9 | | 3.19 | | Proteasome subunit | | |
| IPI00028004 | proteasome subunit, beta type, 3 | 31.7 | 5 | | 3.27 | | Proteasome subunit | | |
| IPI00384051 | proteasome activator complex subunit 2 | 48.1 | 8 | | 4.06 | | Proteasome subunit | | |
| IPI00219622 | proteasome subunit, alpha type, 3 | 51.3 | 11 | | 4.83 | | Proteasome subunit | | |
| IPI00479722 | proteasome activator complex subunit 1 | 36.5 | 8 | | 6.36 | | Proteasome subunit | | |
| IPI00000783 | proteasome subunit, beta type, 8 | 20.6 | 6 | | 6.51 | | Proteasome subunit | | |
| IPI00029623 | proteasome subunit, alpha type, 6 | 32.9 | 8 | | 7.34 | | Proteasome subunit | | |
| IPI00027933 | proteasome subunit, beta type, 10 | 19 | 5 | | 9.84 | | Proteasome subunit | | |
| IPI00000787 | proteasome subunit, beta type, 9 | 13 | 2 | | 40 | | Proteasome subunit | | |

§ Fold change of 40 indicates that the protein is unique in fatal CM.
