# Supplementary material for: Proteomic profiling of the plasma of Gambian children with cerebral malaria
Source: Malar J. 2018 Sep 24;17:337. doi: 10.1186/s12936-018-2487-y (PMC6154937; doi:10.1186/s12936-018-2487-y)
Supplement: Supplementary file 2 — Additional file 2. Circulating proteasomes and PSMB9 in patients with cerebral malaria. Dot plots show the concentration of total circulating 20S proteasomes (left panels) and PSMB9 (right panels) in children with CM based on outcome (top), presence of seizures during admission (middle) and the correlation of these biomarkers with the number of seizures witnessed during admission (bottom). [file 12936_2018_2487_MOESM2_ESM.docx]

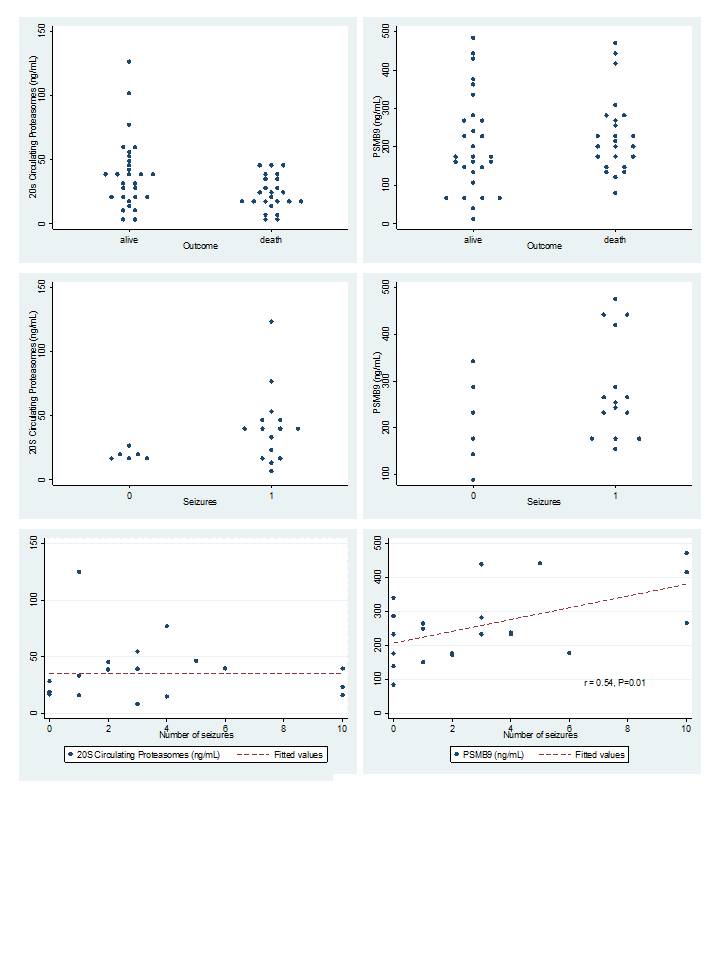


**Additional File 2: Circulating proteasomes and PSMB9 in patients with cerebral malaria.** Dot plots show the concentration of total circulating 20S proteasomes (left panels) and PSMB9 (right panels) in children with CM based on outcome (top), presence of seizures during admission (middle) and the correlation of these biomarkers with the number of seizures witnessed during admission (bottom).
